# Mapping the interview transcript: Identifying spatial policy areas from daily working practices

**DOI:** 10.1111/area.12408

**Published:** 2017-11-24

**Authors:** Scott Orford, Brian Webb

**Affiliations:** ^1^ School of Geography and Planning Cardiff University Wales Institute of Social and Economic Research, Data and Methods (WISERD) Cardiff UK; ^2^ School of Geography and Planning Cardiff University Cardiff UK

**Keywords:** GIS, interview transcripts, localities, mapping, spatial policy, Wales

## Abstract

An interview transcript can be a rich source of geographical references whose potential are not always fully realised in their conventional analysis. Geo‐referencing techniques can be used to assign a spatial footprint to place names, adding value to these data and allowing the geographic information within them to be exploited when coupled with GIS technology. This paper discusses a method of analysing and visualising interview transcripts in order to understand the spatial extent of public policy practitioners’ activities. Through aggregation and statistical mapping it is possible to gain insight into the importance of space across a range of public policy themes and to understand the relationship between practitioner‐defined policy themes and the formal administrative boundaries within which they typically work. The research demonstrates that spatial working practices rarely conform to formal administrative boundaries and that there are varying degrees of spatial focus between different policy themes within localities. It also reveals that spatial working practices can continue to be influenced by historic geographies and can be pulled in different directions, reflecting both the devolved nature of the sector and the particular geographical context of the setting. It concludes that mapping the interview transcript can add value and provide additional insights to more conventional analysis.

## 1 INTRODUCTION

An interview transcript can often be a rich source of geographical references whose potential are not always fully realised in their analysis. Originally developed as a means to manage and analyse quantitative data, the last decade has seen attempts to expand the use of GIS towards more qualitative, particularly textual, data management and analysis (Gregory & Hardie, 2011; Jung & Elwood, 2010; Kwan & Knigge, 2006). Geo‐referencing techniques, for example, have been used to assign a spatial footprint to place names, adding value to these data and allowing the geographic information within them to be exploited when coupled with GIS technology (Goldberg, 2011; Gregory & Hardie, 2011; Wilson et al., 2004). In this respect, proponents of Qualitative GIS have emphasised the power inherent in its geo‐visualisation capabilities to promote and justify its use (Jones & Evans, 2012; Jung & Elwood, 2010; Knigge & Cope, 2006; Knigge & Cope, 2009; Kwan, 2002, 2004; Yuan, 2010).

The underlying purpose for unlocking the geospatial potential of an interview transcript is that the spatial dimension ought to provide additional insight into the understanding and interpretation of the text. Flyvberg and Richardson (1998) have highlighted the significance of understanding the ways in which individuals construct space differently and how multiple concepts of space can often co‐exist and overlap the same geographical space. By mapping qualitative interviews it may be possible to understand, at a fixed point in time, not only the tensions between informal interviewee defined areas and formal geographical boundaries but also how interviewees from different backgrounds conceptualise the space that they live and work in.

**Figure 1:**
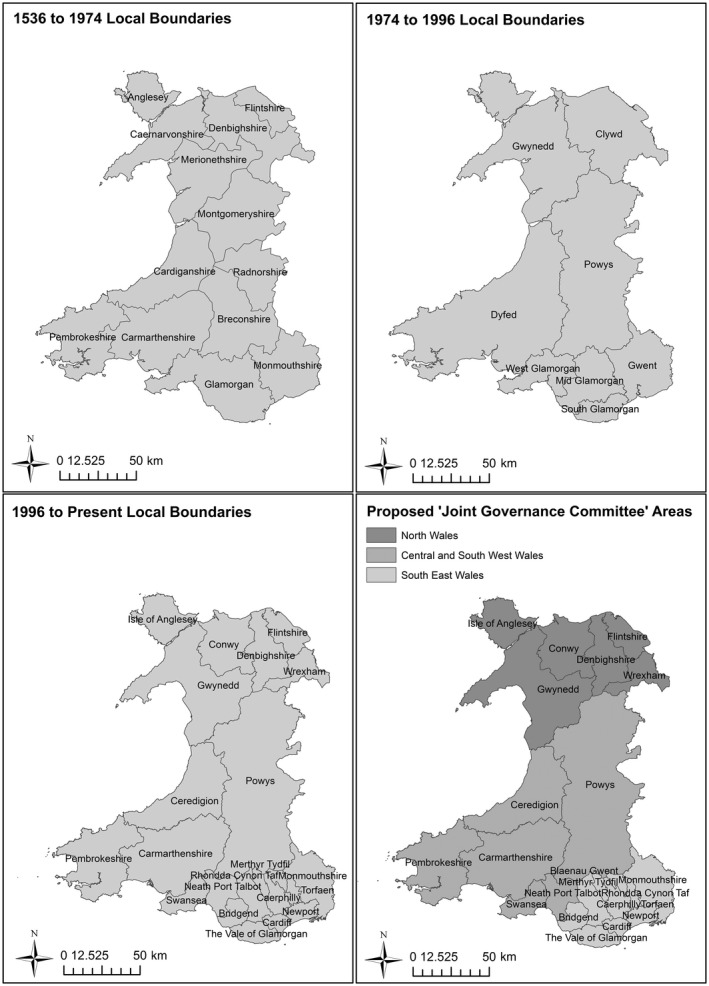
Various administrative boundary changes in Wales. *Source*: Contains Ordnance Survey data © Crown copyright and database right 2012

The aim of this paper is to discuss a method of analysing and visualising interview transcripts in order to understand the spatial extent of local public policy practitioners’ activities in Wales, United Kingdom. A devolved nation comprised of three million people, Wales consists of a mix of highly rural areas to the West and Central parts of the country and more urban areas centred along the South and North of the country linked to England. Historically Wales was divided into 13 local counties from 1536 to 1974, which were then reduced to eight counties until their abolition in 1996 when they were superseded by 22 single‐tier authorities. In 2004, the Welsh Assembly Government (WAG) published the Wales Spatial Plan (WSP), which was designed to improve policy‐making across Wales and facilitate policy integration (Harris & Hooper, 2006). It was promoted as a general framework for collaborative working among the WAG, local authorities and other sectors and it divided Wales into six sub‐regions demarcated by fuzzy boundaries which did not follow pre‐existing administrative boundaries. These fuzzy boundaries caused “initial consternation” among local authority practitioners and they became “less fuzzy in practice” and eventually “firmed up” around existing boundaries (Haughton et al., 2010, p. 146). In 2014, the Williams Commission suggested local authorities be reduced to eight or nine. This proposal was ultimately scraped due to opposition but was followed in 2017 by a Welsh Government White Paper proposing three “joint governance committee” areas to enable local collaboration. Figure 1 summarises these different administrative boundaries within Wales.

Through aggregation and mapping of interview transcripts it is possible to gain insight into the potential importance of these historical, current and proposed boundaries, across a range of local public policy themes, including policing, education, language and culture, environment, health, economic development, housing and employment, and to understand the tension between the daily spaces within which practitioners conduct themselves and the extent to which the administrative areas they are employed within constrain their activities. To do this, the paper is divided into five further sections. The second section provides a discussion of the role of the interview transcript as a spatial representation of individual practices. The third section concerns the process of data collection and geo‐referencing of the text; the fourth section discusses mapping in the context of the qualitative research and policy context of Wales; and the final section provides a conclusion.

## 2 THE INTERVIEW TRANSCRIPT AS A SPATIAL REPRESENTATION

Examples of research that map and spatially analyse large amounts of textual data have emerged over the past few years. Often these are related to historical documents (Knowles, 2008), literary documents (Cooper & Gregory, 2011) or Web 2.0 social media content such as twitter feeds (Field & O'Brien, 2010; Twaroch & Jones, 2010) and the so‐called neogeographies of the GeoWeb (Haklay et al., 2008). The research described here is somewhat different in that it concerns formally collected primary research in the form of interview transcripts of policy practitioners rather than secondary textual data in the form of social media content or documents published in the public domain.

As a record, an interview transcript can provide an individual account of the everyday working practices of participants (McCoy, 2006). These everyday practices have inherent spatial characteristics as interviewees situate their movements, interactions and areas of interest or concern to the interviewer through words (Jones & Evans, 2012; Matthews et al., 2005). Unlike traditional maps, those derived from these narratives may be informed by but are not predicated on fixed boundaries, but rather are fluid, contingent and relational (Thrift, 2007). Maps derived from a lived experience therefore shift the focus away from constraining, predefined and historically embedded geographies and towards an emphasis on process and practice (Harrison, 2000). By making these mundane cartographies visible, we can better understand the spatial scope of individual interactions as they undertake their daily work while making apparent the tensions between formal administrative boundaries and the informal spaces of practice (Amin, 2002; Gerlach, 2014; Jessop, 2000).

Understanding these relationships and tensions is important as conceptions of bounded space continue to play a key role in how individuals conduct themselves in practice (Jones & MacLeod, 2004; MacLeod & Jones, 2007; Tomaney, 2007). The continued influence of bounded space extends to the realm of politics and government, where “in some respects politics in practice still seems to retain a strong territorial focus, or at least territory seems still to provide a significant focus around which a range of political projects are organised” (Cochrane, 2012, p. 95). Martin Jones recognises this in his conceptualisation of phase‐space, an approach that he suggests “acknowledges the relational making of space but insists on the confined, connected, inertial, and always context‐specific nature of existence and emergence” (Jones, 2009, p. 489). He argues that relational thinking does not properly take into account the roles played by boundedness, inertia, power and time. Jones goes on to suggest a phase‐space approach that complements Jessop et al.'s (2008) proposed multidimensional territories, places, scales, networks (TPSN) framework, arguing that the “geography of different moments and their combinations within these spatial constellations merit more systematic phase space “mapping” analysis, tracing continuities and discontinuities” (Jones, 2009, p. 501). In this paper we seek to contribute to Jones’ (2009, p. 500) desire for “a bit of fieldwork” into how to “schematically or diagrammatically “represent” phase space” for the purpose of developing a “geographical narrative” of the daily working practices of different policy practitioners in Wales in order to assess the historical influence of institutional boundaries while identifying overlapping, alternative and emergent definitions of space.

## 3 MAPPING INTERVIEW TRANSCRIPTS TO IDENTIFY PUBLIC POLICY AREAS

The research was undertaken as part of a wider localities study by the Wales Institute of Social and Economic Research, Data and Methods (WISERD). The study focused on three regions of Wales – the Heads of the Valleys region north of Cardiff (known as the Cardiff locality) in south‐east Wales; the Central and West Coast region (comprising the unitary authorities of Ceredigion and Pembrokeshire and the former district of Montgomeryshire in Powys and known as the Aberystwyth locality); and the A55 corridor from Wrexham to Holyhead in North Wales (known as the Bangor locality). The research involved interviewing 120 public policy practitioners across the three localities who have links to one of eight policy themes identified by the Welsh Government as reflecting the range of key devolved and non‐devolved policy areas that the Welsh Government and Local Authorities were organised around at the time of the research. These were loosely based around the 20 policy fields listed in Schedule 5 of the Government of Wales Act 2006: Crime, public space and policing; Language, citizenship and identity; Health, wellbeing and social care; Housing and transport; Education and young people; Environment, tourism and leisure; Economic development and regeneration; and Employment and training.

To identify the practitioners, two unitary authorities in the Aberystwyth (Ceredigion, Pembrokeshire) and Bangor (Gwynedd, Wrexham) localities and three unitary authorities (Blaenau Gwent, Merthyr Tydfil, Rhondda Cynon Taf) in the Cardiff locality were selected and the practitioners mapped across the seven unitary authorities by their role in the organisations (Table 1). The majority of the practitioners were based within a unitary authority, but some worked for organisations that cut across authorities, such as the Health Service or Environment Agency, and these are identified as “other.”

**Table 1:** A summary of the practitioner interviews by unitary authority

Locality	Unitary authority	Number of interviewees
Aberystwyth	Ceredigion	13
	Pembrokeshire	10
	Other	13
Bangor	Gwynedd	22
	Wrexham	13
	Blaenau Gwent	11
Cardiff	Merthyr Tydfil	10
	Rhondda Cynon Taf	20
	Other	8

The focus of the interviews was on the role of the practitioners within their policy area and how their understanding of their “patch” – a spatial term deliberately chosen for its potential broad and flexible interpretation by individuals (Jones et al., 2013) – in which they worked influenced what they did. Hence the interviews had a strong geographical slant with the interviewees encouraged to discuss the places important in their work. Transcripts were made, coded and analysed by qualitative researchers using a Computer Assisted Qualitative Data Analysis Software (CAQDAS) package. As part of the coding, a researcher went through all the transcripts and identified place names (toponyms) within them. These place names were then geo‐referenced to a single point using the Ordnance Survey (OS) 1:50,000 scale gazetteer (OS, 2010).

The place names were geo‐referenced following a four‐stage process. In the first stage the place names were identified in the transcript manually by one of the researchers. The second stage automatically matched the place names against place names in the OS 1:50,000 scale gazetteer which had been edited so that it only contained place names in Wales. The third stage concerned the manual disambiguation of multiple matches of a single place name in the transcript to more than one place name in the gazetteer. In the final stage, unmatched place names were manually matched with the OS gazetteer or matched against other references containing Welsh toponyms (see Table 2). The process was complicated by the bilingual nature of Welsh place names, which resulted in some inconsistencies in spellings and usage within and between the transcripts and the gazetteer. This was exacerbated by inaccuracies introduced during transcription where the (typically non‐Welsh‐speaking) transcriber looked‐up or sometimes guessed at the spelling of less common place names, resulting in spelling errors (for a recent commentary on the issues of matching Welsh place names to OS address products, see Fry et al., 2017). However, there did not seem to be any evidence of conflict in the use of Welsh place names in the daily working practices of interviewees, even in English‐speaking areas of the localities, possibly because of the institutionalised nature of the Welsh language in the organisations in which the practitioners worked.

**Table 2:** A summary of the geo‐referencing process and the success of matching at different stages

Stage	Matching process	Number of place names
Stage 1	Place name identification	8629
Stage 2 Unique matches	Automated one‐to‐one	2231
Stage 3 Ambiguous (Multiple matches)	Manual	359
Stage 4 Unmatched	Other Welsh toponym sources	4664
	Outside of Wales	991
	Vernacular geographies	173
	Manual match	140
	No matches	71

## 4 CONSTRUCTING STATISTICAL MAPS

Mapping of place names through dot maps or density surfaces is useful for noting the frequency or concentration of spatial references, however such an approach is limiting. Dot maps can appear cluttered and difficult to interpret, while plotting individual locations can be potentially disclosive as it may allow the identification of the interviewee or the subjects of the interview. Outliers can also provide potentially false impressions of typical spatial interactions. Density surfaces may be less disclosive but can suffer from arbitrarily defined bandwidths, resulting in maps with different degrees of smoothing. Rather than use dot maps and density surfaces, more appropriate methods of mapping the spatial extent of policy areas potentially may include the use of centrographic techniques (Alexander et al., 2011; Vanhulsel et al., 2011). In this approach, traditional point locations are replaced by visual statistical summaries of the locations such as standard deviational ellipses and mean centres. A one standard deviational ellipse represents approximately 68% of the points and is centred on the mean centre of the point pattern, with its long axis in the direction of the maximum dispersion and its short axis in the direction of the minimum dispersion. Hence an ellipse is produced if the points have a directional component, otherwise the ellipse will be more or less circular. As the spatial ellipses may overlap, it is possible to extract an area that is common to all the policy themes and define this as the “core area” or main spatial focus of working practices in that locality. The ellipses and core areas were calculated using ArcGIS 10.3.

### 4.1 The Aberystwyth locality

**Figure 2:**
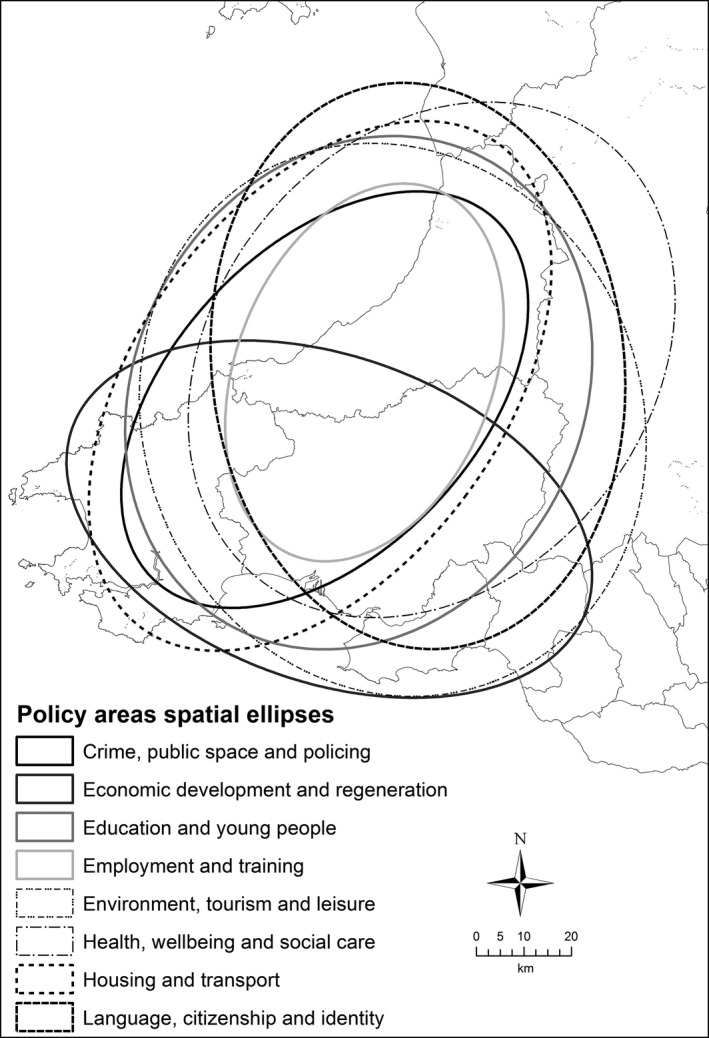
Spatial ellipses of the Aberystwyth locality practitioner interviews by policy themes. *Source*: Contains Ordnance Survey data © Crown copyright and database right 2012

**Figure 3:**
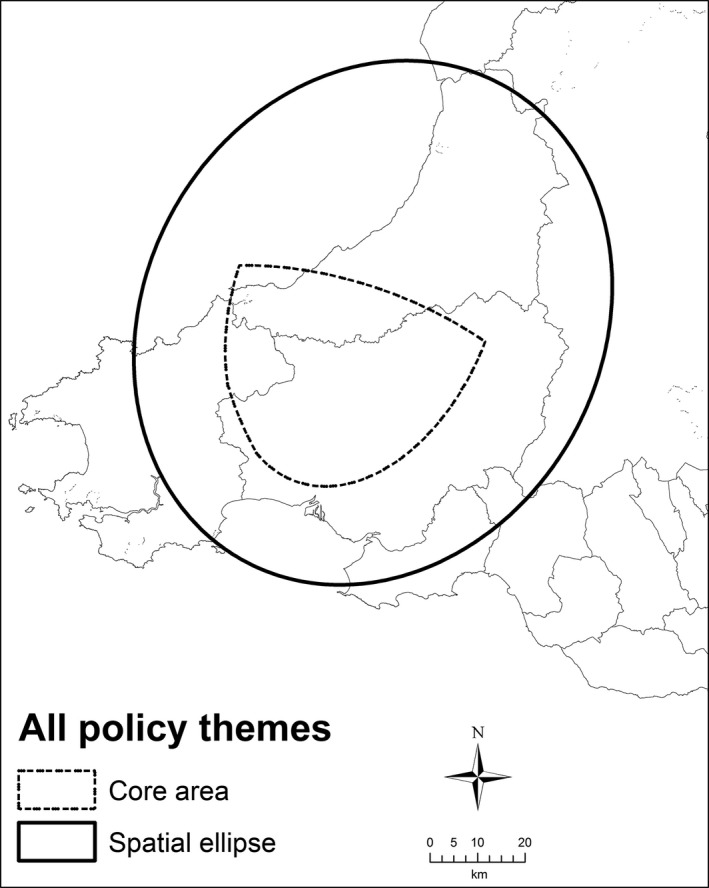
Areas in common in the Aberystwyth locality practitioner interviews. *Source*: Contains Ordnance Survey data © Crown copyright and database right 2012

Figure 2 shows the spatial ellipses for the places mentioned in the Aberystwyth transcripts categorised by policy theme. There is a strong north‐east–south‐west orientation of the ellipses covering Carmarthenshire, Ceredigion and north‐west Pembrokeshire. This spatial focus is interesting because it encompasses the former county of Dyfed, which was replaced in the 1996 Welsh local government reorganisation and therefore could reflect the continued legacy of past administrative organisational structures, which was not directly evident in an analysis of the interview transcripts. The spatial ellipses of two policy areas – Language, citizenship and identity and Economic development and regeneration – have noticeably different orientations. The former is north–south and notably does not include the predominately English‐speaking communities of Pembrokeshire; the latter has an east–west orientation focused on Swansea and the authorities to the east. Analysis of the interview transcripts for this ellipse suggests that the majority of regeneration policies are aimed at the former industrial and mining communities located here. The largest ellipse is for environment, tourism and leisure, reflecting the dependence on tourism as a key economic activity across the whole of the locality. The smallest ellipse is for crime, public space and policing, with the interviews revealing this being down to both the un‐devolved nature of the policy field and the spatial focus on particular problem communities. Figure 3 shows the spatial policy areas common to all themes. The core area clearly highlights the territory controlled by the former Dyfed council and again suggests that despite being split between three unitary authorities, the working practices of the stakeholders in the locality are strongly influenced by former administrative boundaries. The spatial ellipse of all the stakeholders covers a much wider area but does not include anywhere north of Ceredigion, perhaps reflecting the influence of Swansea and the Valleys in the south on the policy focus of the locality.

### 4.2 The Bangor locality

**Figure 4:**
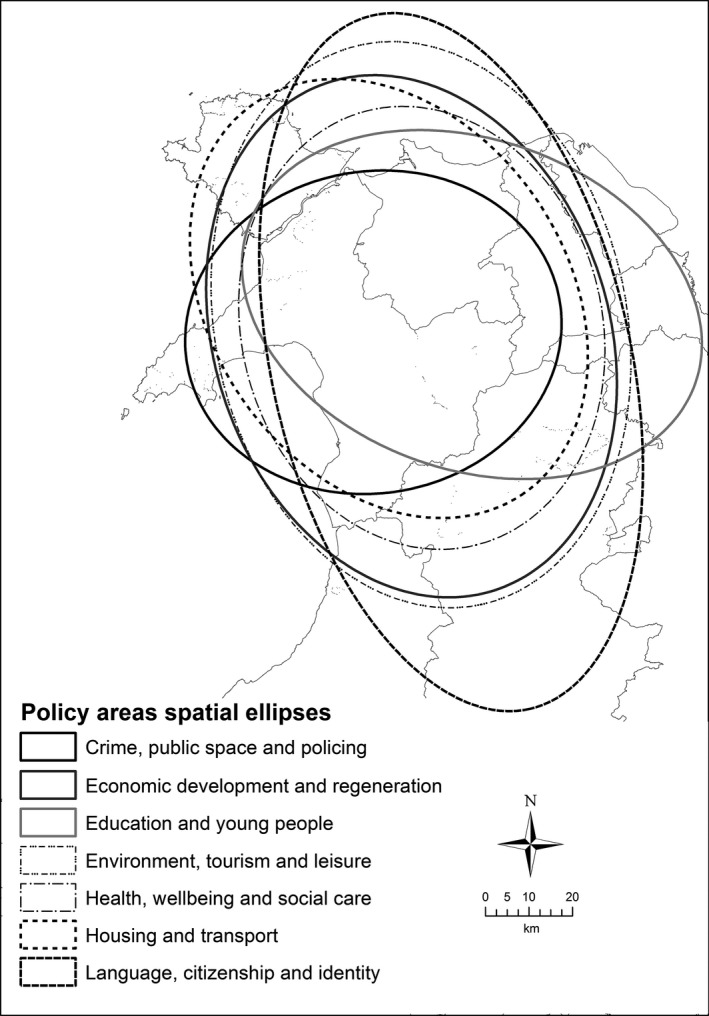
Spatial ellipses of the Bangor locality practitioner interviews by policy themes. *Source*: Contains Ordnance Survey data © Crown copyright and database right 2012

**Figure 5:**
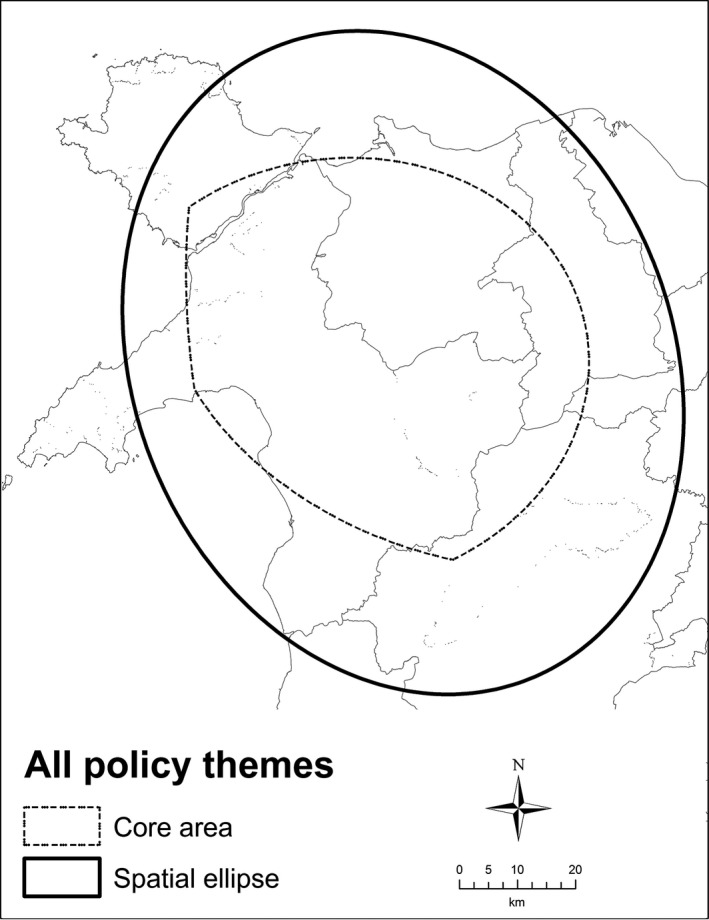
Areas in common in the Bangor locality practitioner interviews. *Source*: Contains Ordnance Survey data © Crown copyright and database right 2012

Figure 4 contains the spatial ellipses for the seven policy themes in the Bangor locality, revealing that the spatial focus of the stakeholder working activities go beyond north Wales and the A55 corridor and extend south into mid‐Wales. Indeed, the general orientations of the ellipses are north–south rather than east–west, suggesting that the principal transportation routes in the locality are not as influential in structuring the working practices of the stakeholders as ties with mid‐ and south Wales. This goes against the tacit understanding that the locality is significantly influenced by east–west flows along the coastal region and also some of the findings from the interview transcripts. Two policy areas that do exhibit a strong east–west orientation in their spatial focus are Education and young people and Crime, public space and policing, which may reflect cross‐border relations with England in these policy themes and in practitioner working practices. The largest ellipse was for Language, citizenship and identity, reflecting the importance of the Welsh language in this part of Wales, with interviews highlighting problems caused by in‐migration and second home ownership. Similar to Aberystwyth, the smallest ellipse was for Crime, public space and policing. Figure 5 shows that the core policy area includes the Snowdonia National Park and Conwy. It does not cover Anglesey or Wrexham, or extend to the Welsh border. The All Policy ellipse is similar in shape and orientation but larger and encompasses the majority of North Wales and north Powys, again indicating the links to Wales to the south rather than to England to the east.

### 4.3 The Cardiff locality

**Figure 6:**
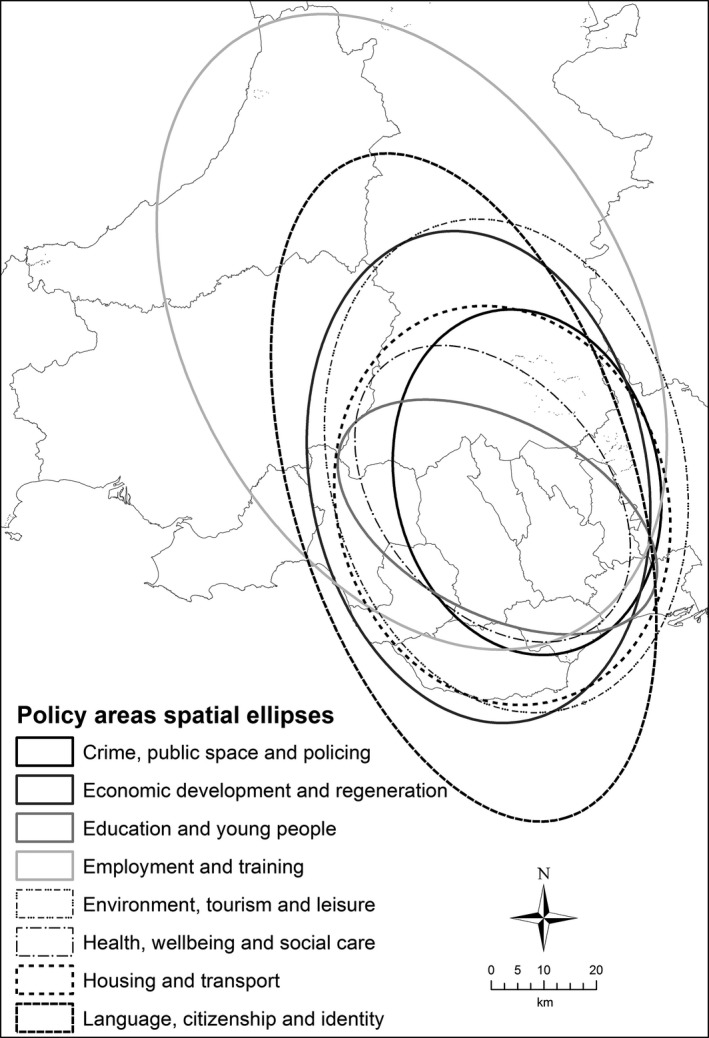
Spatial ellipses of the Cardiff locality practitioner interviews by policy themes. *Source*: Contains Ordnance Survey data © Crown copyright and database right 2012

**Figure 7:**
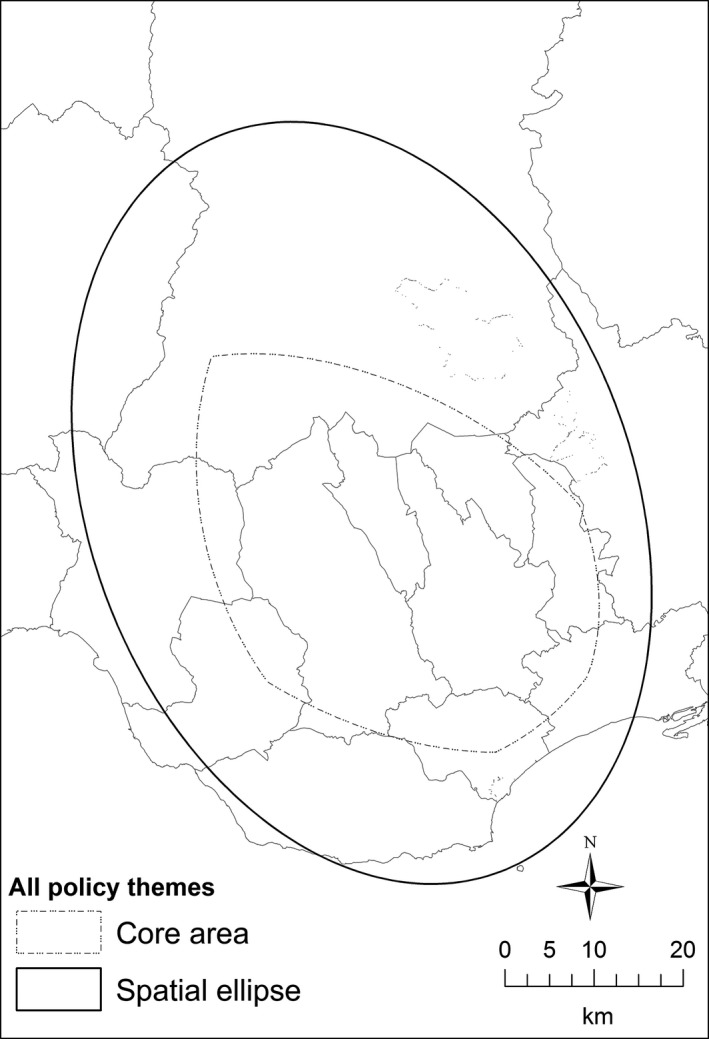
Areas in common in the Cardiff locality practitioner interviews. *Source*: Contains Ordnance Survey data © Crown copyright and database right 2012

Figure 6 shows the spatial ellipses for the stakeholder interviews for each policy theme in the Cardiff locality. Although these are all centred on the Heads of the Valleys, some extend significantly westwards and northwards, suggesting ties and working practices stretching into west and mid‐Wales. Three policy themes fit closely with the Heads of the Valleys: Education and young people; Health, wellbeing and social care; and Crime, public space and policing. The tight spatial focus in these policy areas is understandable, given the longstanding problems of poor health, poor educational attainment and raised incidents of crime in this region which also emerges from an analysis of the interview transcripts. In comparison, the largest ellipse is for the Employment and training policy area, with the interviews pointing towards a longstanding imbalance between high levels of unemployment in the Heads of the Valleys and job vacancies to the south in the Cardiff and Newport city regions, and to the north in Powys. Figure 7 shows that the Heads of the Valleys is central to the work of all the stakeholders, although south Powys and west towards Swansea is also an important spatial focus of practitioners’ activities.

## 5 DISCUSSION

The 1996 re‐organisation of Welsh local authorities, the establishment of the Welsh Government in 1999 and the role of ever‐changing non‐governmental organisations such as the Welsh Development Agency have moulded the policy context in Wales (Stafford, 2016). It is this background within which the policy ellipses can be understood. Devolution originally transferred limited secondary and executive functions from Westminster to Wales, resulting in a “patchwork of powers” and an administratively complex and fragmented policy arrangement (Rawlings, 2003). Cardiff, as the seat of the devolved government, was at the centre of this patchwork of powers and has inherited some of the powers of the Welsh local authorities in devolved policy areas. The small size of Wales meant that much closer working relationships and stronger vertical links were possible between officials in Cardiff and practitioners at the local level (Stafford, 2016). A good example of this is in the Bangor locality, which encompasses the local authorities that are the furthest from Cardiff geographically, but which has a clear north–south orientation in the spatial ellipses of policy areas that have had the largest transfer of responsibility from Westminster to Cardiff, such as in Employment and Regeneration, and Health and Wellbeing. Here the influence of Cardiff would appear to weaken the practitioner ties to jurisdictions in England to the east, which arguably have more of an immediate impact on the locality in terms of spill‐over effects. The smaller and more focused spatial ellipses of non‐devolved policy areas such as Crime and Policing reveals the practitioner focus on the locality when the links with officials in Cardiff are less important.

However, it is important not to over emphasise the importance of Cardiff in the functioning of local authorities post‐devolution. As Stafford (2016) observes, partnership with local authorities was integral to the development of the new devolved institutions of Welsh Government and in particular the implementation of Welsh policy. Practitioners in local authorities were the experts in implementing policy and the Welsh Government drew on this experience, devolving service delivery to the local level and driving improvement from the bottom up. This is reflected in the design and ambition of the WSP, with local‐level practitioners compelled to collaborate with partners and agencies across administrative boundaries. Although the general agreement is that the fuzzy boundaries in the WSP caused confusion at the local level, the overlapping but distinct geographies of the spatial ellipses suggest that practitioners’ daily working practices go beyond the confines of fixed administrative boundaries and indeed could be defined as fuzzy in the sense that they can be used to define core and peripheral areas of working that do not match their official job demarcations. Indeed, the firming up of the fuzzy boundaries in the WSP was driven by the practical need to use official boundaries for statistical and data reporting purposes as opposed to the implementation of policy and service delivery on the ground (Haughton et al., 2010). The spatial ellipses suggest that it may be conducive to use fuzzy boundaries in providing a framework for policy implementation, but these should be empirically informed and reflect actual working practices of those delivering services at the local level. As Stafford (2016) reflects, the policy context of Wales is still highly influenced by pre‐devolution agencies, institutions and organisation, illustrated by the continuing influence of the former county Dyfed in the Aberystwyth locality, and this should be acknowledged in future planning and policy frameworks in Wales such as the 2017 proposed joint governance committee areas.

## 6 CONCLUSION

This paper has discussed a methodological approach to spatialising transcript data based on interviews with public policy practitioners. This has allowed an analysis of the importance of particular locations for public policy practitioners, demonstrating the potential of this approach to aid understanding of the spatial relationships of each policy sector and their potential spheres of influence. It allows for an improved understanding of the tensions between bounded, administrative spaces and the more abstract understandings of space derived from practice (Gerlach, 2014). These tensions are not always directly evident from a reading of the interview transcripts. The maps have illustrated the everyday spatial working practices of stakeholders in different policy themes. By making the mundane cartographies of their working practices visible, we have revealed the spatial scope of interactions between different policy themes and the relationships between formal territories and informal spaces. Returning to Jones’ (2009) conceptualisation of phase‐space in relational thinking, this “bit of fieldwork” has demonstrated the continuities and discontinuities of the spatial extent of practitioner working practices with regard to pre‐ and post‐devolution Wales. Continuities are reflected in the importance of historic ties of former administrative organisations – long since abolished but still prevalent in the traces made by the spatial ellipses. These also point towards the possible inertia of daily working practices, which are difficult to change even with the implementation of new geographic regimes. Discontinuities are evident in the importance of Cardiff post‐devolution, forging new collaborations and orientating working practices towards the south‐east of the country, although still retaining a local focus. Fixed boundaries still have an important role to play, but fuzziness is also evident in the overlapping and divergent geographies of the spatial ellipses that cross local authority and other boundaries. The size, shape and location of the core areas demonstrat the varying degree of spatial focus of the different policy themes between the localities. This can also be seen in the overlap, or lack of overlap, of the different policy ellipses providing insight into practitioners’ daily experience of space and place when working in their locality.
